# A distinct repertoire of cancer‐associated fibroblasts is enriched in cribriform prostate cancer

**DOI:** 10.1002/cjp2.205

**Published:** 2021-02-18

**Authors:** Amanda B Hesterberg, Brenda L Rios, Elysa M Wolf, Colby Tubbs, Hong Yuen Wong, Kerry R Schaffer, Tamara L Lotan, Giovanna A Giannico, Jennifer B Gordetsky, Paula J Hurley

**Affiliations:** ^1^ Department of Medicine Vanderbilt University Medical Center Nashville TN USA; ^2^ Department of Pathology Johns Hopkins School of Medicine Baltimore MD USA; ^3^ Department of Pathology Vanderbilt University Medical Center Nashville TN USA; ^4^ Department of Urology Vanderbilt University Medical Center Nashville TN USA; ^5^ Vanderbilt‐Ingram Cancer Center Vanderbilt University Medical Center Nashville TN USA

**Keywords:** cribriform, prostate, cancer, cancer‐associated fibroblasts, ASPN, FAP, THY1, ENG, NT5E, PDGFRβ, tumor microenvironment, stroma, RNAscope

## Abstract

Outcomes for men with localized prostate cancer vary widely, with some men effectively managed without treatment on active surveillance, while other men rapidly progress to metastatic disease despite curative‐intent therapies. One of the strongest prognostic indicators of outcome is grade groups based on the Gleason grading system. Gleason grade 4 prostate cancer with cribriform morphology is associated with adverse outcomes and can be utilized clinically to improve risk stratification. The underpinnings of disease aggressiveness associated with cribriform architecture are not fully understood. Most studies have focused on genetic and molecular alterations in cribriform tumor cells; however, less is known about the tumor microenvironment in cribriform prostate cancer. Cancer‐associated fibroblasts (CAFs) are a heterogeneous population of fibroblasts in the tumor microenvironment that impact cancer aggressiveness. The overall goal of this study was to determine if cribriform prostate cancers are associated with a unique repertoire of CAFs. Radical prostatectomy whole‐tissue sections were analyzed for the expression of fibroblast markers (*ASPN* in combination with *FAP*, *THY1*, *ENG*, *NT5E*, *TNC*, and *PDGFRβ*) in stroma adjacent to benign glands and in Gleason grade 3, Gleason grade 4 cribriform, and Gleason grade 4 noncribriform prostate cancer by RNAscope®. Halo® Software was used to quantify percent positive stromal cells and expression per positive cell. The fibroblast subtypes enriched in prostate cancer were highly heterogeneous. Both overlapping and distinct populations of low abundant fibroblast subtypes in benign prostate stroma were enriched in Gleason grade 4 prostate cancer with cribriform morphology compared to Gleason grade 4 prostate cancer with noncribriform morphology and Gleason grade 3 prostate cancer. In addition, gene expression was distinctly altered in CAF subtypes adjacent to cribriform prostate cancer. Overall, these studies suggest that cribriform prostate cancer has a unique tumor microenvironment that may distinguish it from other Gleason grade 4 morphologies and lower Gleason grades.

## Introduction

Localized prostate cancer is heterogeneous for disease aggressiveness that ranges from indolent cancers not needing treatment to highly aggressive cancers requiring multimodal therapies. One of the strongest prognostic indicators of disease aggressiveness and a critical diagnostic parameter for clinical decision‐making is grade groups [1, 2, 3], a modified system based on the prior Gleason grading system [4]. Studies suggest that risk may be further stratified by the presence of cribriform morphology, one of the four major histologic subtypes of Gleason pattern 4 prostate cancer that also includes fused, poorly formed, and glomeruloid [5]. As a subtype of Gleason 4, cribriform morphology spans grade groups 2–5 and can be present either diffusely or focally. Cribriform morphology has been associated with adverse clinicopathologic findings and outcomes [6, 7, 8, 9, 10, 11, 12]. Gleason grade 4 prostate cancer with cribriform morphology at biopsy was associated with higher tumor grade and stage at time of curative‐intent surgery [6]. Even within a grade group, men who have prostate cancer containing cribriform morphology are more likely to have adverse outcomes. In radical prostatectomy specimens, cribriform morphology was independently associated with worse biochemical recurrence‐free and metastasis‐free survival in men with grade group 4 (Gleason score 8) prostate cancer [7]. Cribriform prostate cancer was also associated with progression to lethal prostate cancer, independent of Gleason score [13]. The exact prevalence of cribriform prostate cancer is uncertain due to inconsistent reporting of cribriform morphology [14], undersampling during biopsy [6, 15], bias toward intermediate‐ and high‐risk Gleason grade cases in radical prostatectomy cohorts [16], and lack of distinction between cribriform and intraductal carcinoma [17]. However, a recent report detected cribriform morphology in 34% of prostate cancer biopsies [18], supporting that a substantial number of patients has this subtype of Gleason grade 4 prostate cancer. Collectively, these studies suggest that cribriform morphology may have clinical utility in the risk stratification of some patients.

The genetic and molecular factors driving worse outcomes in patients with Gleason grade 4 prostate cancer with cribriform morphology are beginning to be elucidated. Recent studies support that cribriform prostate cancers are enriched with distinct genetic and epigenetic alterations even when compared to other Gleason grade 4 morphologies. Analyses of The Cancer Genome Atlas and the Canadian Prostate Cancer Genome Network support that prostate cancers with cribriform architecture have increased genetic instability and copy number alterations [13, 19, 20] with frequent genetic alterations to known drivers of prostate cancer including *PTEN* and *SPOP* [13, 19]. Dysregulation of MYC proto‐oncogene, bHLH transcription factor (MYC) [13, 19], mammalian target of rapamycin complex 1 (mTORC1) [13], mitogen‐activated protein kinase (MAPK) [13], Janus kinase ‐ signal transducer and activator of transcription (JAK‐STAT) [13], and epidermal growth factor receptor (EGFR) [21] expression and/or pathways has also been shown to be enriched in cribriform prostate cancer. In addition to protein‐coding genes, cribriform prostate cancers often have increased expression of *SChLAP1* [20], a long noncoding RNA associated with prostate cancer progression to metastasis [22, 23]. These studies suggest that cribriform prostate cancers may have distinct molecular repertoires that promote their aggressive phenotype.

In addition to carcinoma‐specific features, the tumor microenvironment influences tumor development and progression [24]. A key component of the tumor microenvironment is the heterogeneous population of fibroblast‐like cells, which are collectively referred to as cancer‐associated fibroblasts (CAFs) [25, 26]. CAF heterogeneity may vary widely by cancer type, grade, and stage. CAFs have dynamic and pleiotropic roles in the tumor microenvironment, with certain subtypes likely functioning to restrict, while others function to drive cancer progression [27, 28]. Cribriform prostate cancer has a distinct morphology recently described as *nimbosus* (Latin for gathering of stormy clouds) [20]. A hallmark of this architecture is that most cribriform cancer cells are not in direct contact with the often highly abundant surrounding CAFs. Little is known about CAF markers or distinct CAF subtypes adjacent to cribriform prostate cancer or how they differ compared to CAFs associated with other Gleason grade 4 morphologies. If differences exist, it is not known if they differentially impact tumor progression.

Asporin (ASPN) is enriched in prostate CAFs but not in other activated fibroblasts such as those associated with prostate inflammation [29, 30, 31, 32]. Studies support that ASPN has key roles in modifying the tumor microenvironment and promoting tumor progression to metastasis [29, 30, 31, 32, 33, 34]. ASPN expression in the tumor microenvironment is associated with increasing Gleason grade and worse outcomes [29, 30, 31, 32, 33], but how ASPN expression in CAFs adjacent to Gleason grade 4 prostate cancer with cribriform morphology compares to CAFs in Gleason grade 4 prostate cancer with noncribriform morphology is not known. Furthermore, it is not known if increased ASPN expression in the tumor microenvironment is due to increased ASPN^+^ cells, possibly to increased ASPN expression per cell, or a combination of both. It is also not known if elevated ASPN is due to the expansion of a uniform subtype of fibroblasts or a heterogeneous population of ASPN^+^ cells.

The CAF subtypes that express ASPN, as well as the coexpression of ASPN with other CAF markers, have not been fully delineated due to the secreted nature of ASPN. Fibroblast activation protein‐α (FAP), a canonical CAF marker, is similarly associated with worse outcomes [35, 36]. FAP regulates cellular migration, angiogenesis, and immune suppression [37, 38, 39, 40], and it is a candidate CAF target for multiple therapeutic and imaging strategies [41, 42, 43, 44, 45, 46]. Similar to FAP, Thy‐1 cell surface antigen (THY1) is also overexpressed in prostate CAFs [47], and it may have a role in regulating cancer stem cells [48]. Endoglin (ENG) is another CAF marker associated with adverse outcomes, and it has been shown to promote castration‐resistant prostate cancer [49, 50, 51]. Tenascin C (TNC), 5'‐nucleotidase ecto (NT5E), and platelet‐derived growth factor receptor beta (PDGFRβ) are other CAF markers that are currently candidate targets for imaging or therapeutics [52, 53, 54, 55]. The expression of these CAF markers in ASPN^+^ cells or in Gleason grade 4 prostate cancer with cribriform morphology is not known.

The overall goal of this study was to determine if cribriform prostate cancer is associated with a unique repertoire of CAFs compared to other Gleason grade 4 morphologies. The primary objective was to determine if CAF markers associated with Gleason grade 4 prostate cancer with cribriform morphology are distinct from Gleason grade 4 prostate cancer without cribriform morphology. The secondary objective was to begin to delineate the heterogeneity of CAF subtypes associated with cribriform prostate cancer. To do this, the RNAscope® Duplex Assay [56] was used to analyze the expression of *ASPN* in the stroma adjacent to benign glands, Gleason grade 3, Gleason grade 4 cribriform, and Gleason grade 4 noncribriform prostate cancer in radical prostatectomy specimens in dual combination with other markers expressed in fibroblast lineage cells: *FAP*, *THY1*, *ENG*, *NT5E*, *TNC*, and *PDGFRβ*.

## Materials and methods

### Patient selection

This study was approved by the Institutional Research Boards at Vanderbilt University Medical Center and Johns Hopkins School of Medicine. Twenty‐one radical prostatectomy cases between 2016 and 2019 with grade group 2–5 prostate cancer were selected and reviewed by a genitourinary pathologist. Whole slides were assessed by RNAscope®. Cases were examined for areas of stroma adjacent to benign prostate (*n* = 21), Gleason 3 prostate cancer (*n* = 13), Gleason 4 noncribriform prostate cancer (*n* = 17), and Gleason 4 cribriform prostate cancer (*n* = 12).

### Dual RNA *in situ* hybridization

Patient formalin‐fixed paraffin‐embedded (FFPE) whole‐tissue sections (4 μm) from radical prostatectomy specimens were analyzed for expression of *ASPN* in combination with the following markers: *FAP*, *THY1*, *ENG*, *NT5E*, *TNC*, and *PDGFRβ* (see supplementary material, Table S1) using the RNAscope® 2.5 HD Duplex Assay by Advanced Cell Diagnostics (322430; ACD, Newark, CA, USA) according to the manufacturer's recommendations (see supplementary material, Supplementary materials and methods, and Tables S1 and S2).

### Immunohistochemistry

Immunohistochemistry (IHC) was performed as described previously [57]. In brief, patient FFPE whole‐tissue sections (4 μm) from radical prostatectomy specimens were deparaffinized; steamed in Antigen Retrieval for 40 min; blocked with Protein Block Serum‐Free (Dako, Agilent Dako, Santa Clara, CA, USA); and then incubated with primary antibodies to ASPN, FAP, THY1, ENG, NT5E, and PDGFRβ (see supplementary material, Table S3) overnight at 4 °C. Primary antibodies were followed by secondary antibodies (Vector Laboratories, Burlingame, CA, USA) and then detected with a 3,3'‐diaminobenzidine kit (Vector Laboratories).

### RNAscope® probe and antibody validation

RNAscope® probe specificity was determined on FFPE cell pellets of HEK‐293 cells (ATCC, CRL‐1573) expressing empty vector, *hASPN* (RC209353; Origene, Rockville, MD, USA), *hFAP* (RG215251; Origene), *hTHY1* (RG209458; Origene), *hENG* (RG226069; Origene), *hNT5E* (RG209568; Origene), *hTNC* (RG215251; Origene), or *hPDGFRβ* (RG206377; Origene) cDNA (see supplementary material, Figure S1A). Lipofectamine 3000 (L300015; Invitrogen, Carlsbad, CA, USA) was used to transfect HEK‐293 cells according to the manufacturer's protocol. HEK‐293 were determined to be negative or very low by RT‐qPCR for the above genes compared to human CAFs (see supplementary material, Figure S1B). RNAscope® technique was also validated by the HeLa control slide (310045; ACD) and corresponding positive and negative probes provided by ACD (321641 and 320751) (see supplementary material, Figure S1C). Antibody specificity was similarly determined (see supplementary material, Figure S2). None of the antibodies tested against TNC passed validation. Control slides for RNAscope® and IHC were analyzed in tandem with patient samples.

### Quantification

Grading of tumor and designation of cribriform morphology was based on current international pathologic guidelines [17]. Slides chosen for histologic evaluation from prostatectomy specimens contained Gleason grade 3 and 4 tumors. Cribriform glands were not excluded based on size. The diagnosis of invasive cribriform prostate cancer was made based on the histologic examination of H&E‐stained slides. Immunohistochemical stains for basal cell markers were not performed to distinguish invasive Gleason grade 4 cribriform tumors from intraductal carcinoma. Whole slides were scanned for brightfield imaging at the Vanderbilt Digital Histology Core (SCN400; Leica, Wetzlar, Germany). Representative areas of stroma adjacent to predominantly benign prostate (*n* = 21), Gleason grade 3 prostate cancer (*n* = 13), Gleason grade 4 noncribriform prostate cancer (*n* = 17), and Gleason grade 4 cribriform prostate cancer (*n* = 12) that were greater than 1 mm apart were identified, and probe staining was analyzed using Halo® Software v3.0.311.328 (Indica labs, Albuquerque, NM, USA). Slides were quantified for the percentage of stromal cells that were positive for individual and dual probe staining. Intensity of probe staining was quantified and then divided by the number of positive stromal cells to determine the relative mean expression per positive stromal cell. The *H*‐score was calculated by the percent positive stromal cells × the relative mean expression per positive stromal cell. Cases that were stained more than once for *ASPN* were averaged for percent *ASPN* ^+^ stroma and *ASPN* expression before inclusion in the final analyses for the case.

### Statistical analyses

Statistical comparisons between multiple groups were performed using one‐way analysis of variance with Tukey multiple comparisons. Statistical significance was defined as a *P* value <0.05, and *P* values are indicated with asterisks in the figures as follows: *p ≤ 0.05; **p ≤ 0.01; ***p ≤ 0.001; and ****p ≤ 0.0001. All statistical comparisons were performed using GraphPad Prism software (v8.0) (San Diego, CA, USA).

## Results

### *ASPN* expression in benign prostate and prostate cancer

IHC and RNAscope® are complementary assays (see supplementary material, Figure S2); however, IHC is limited in its ability to accurately identify the cell of origin for secreted proteins like ASPN [58]. To circumvent this limitation, RNAscope® was used to assess *ASPN* mRNA expression. RNAscope® enables the localization of RNA molecules within a sample, which can then be mapped to a single cell of interest [56]. Whole slides from radical prostatectomy were examined for *ASPN* expression in stroma adjacent to benign prostate (*n* = 21) and in Gleason grade 3 (*n* = 13), Gleason grade 4 without cribriform morphology (*n* = 17), and Gleason grade 4 with cribriform morphology (*n* = 12) prostate cancer (see supplementary material, Table S4).

**Figure 1:**
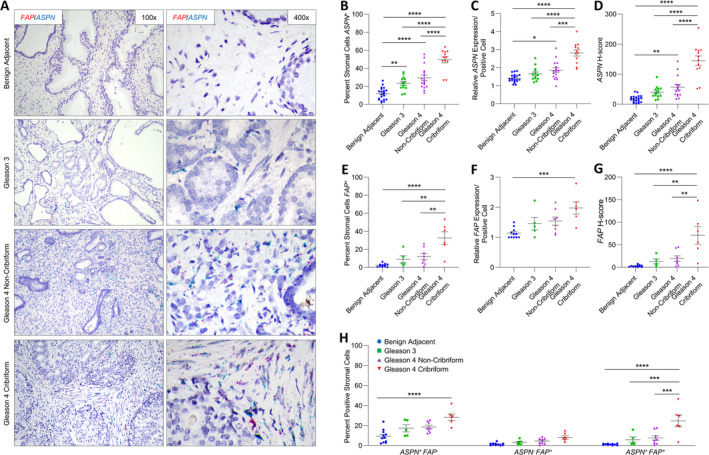
*ASPN* and *FAP* expression in cribriform prostate cancer. (A) Representative images of dual *ASPN* (blue) and *FAP* (red) mRNA expression in stroma adjacent to benign prostate, Gleason grade 3 prostate cancer, Gleason grade 4 prostate cancer without cribriform morphology, and Gleason grade 4 prostate cancer with cribriform morphology (×400 magnification). (B) The percentage of *ASPN* ^+^ stromal cells adjacent to benign prostate (*n* = 21), Gleason grade 3 prostate cancer (*n* = 13), Gleason grade 4 prostate cancer without cribriform (*n* = 17), and Gleason grade 4 prostate cancer with cribriform morphology (*n* = 12). (C) The relative expression of *ASPN* in *ASPN* ^*+*^ stromal cells. (D) The mean *ASPN H*‐score. (E) The percentage of *FAP* ^*+*^ stromal cells adjacent to benign prostate (*n* = 11), Gleason grade 3 prostate cancer (*n* = 5), Gleason grade 4 prostate cancer without cribriform morphology (*n* = 8), and Gleason grade 4 prostate cancer with cribriform morphology (*n* = 6). (F) The relative expression of *FAP* in *FAP* ^*+*^ stromal cells. (G) The mean *FAP H*‐score. (H) The percent *ASPN* ^+^ *FAP* ^−^, *ASPN* ^−^ *FAP* ^+^, and *ASPN* ^+^ *FAP* ^+^ stromal cells adjacent to benign prostate (*n* = 11), Gleason grade 3 prostate cancer (*n* = 5), Gleason 4 prostate cancer without cribriform morphology (*n* = 8), and Gleason grade 4 prostate cancer with cribriform morphology (*n* = 6). Gene expression detected by RNAScope® and quantified with Halo® Software. Statistical analyses performed using one‐way analysis of variance with Tukey multiple comparisons. Graphs shown as mean ± SEM, *p ≤ 0.05, **p ≤ 0.01, ***p ≤ 0.001, ****p < 0.0001.

The percentage of *ASPN* ^+^ cells within the stroma adjacent to prostate cancer was significantly increased compared to stroma adjacent to benign prostate. Within the cancer subtypes examined, the percentage of *ASPN* ^+^ cells was the highest within stroma adjacent to Gleason grade 4 prostate cancer with cribriform morphology compared to Gleason grade 4 prostate cancer with noncribriform morphologies and Gleason grade 3 prostate cancer (Figure 1A,B). Furthermore, *ASPN+* cells adjacent to Gleason grade 4 cribriform prostate cancer expressed significantly higher levels of *ASPN* per cell compared to *ASPN* ^+^ cells adjacent to Gleason grade 4 with noncribriform morphologies or Gleason grade 3 prostate cancers (Figure 1C). Consistent with these findings, stroma adjacent to Gleason grade 4 prostate cancer with cribriform morphology had the highest overall *H*‐score for *ASPN* expression (Figure 1D and supplementary material, Table S5).

### *FAP* expression in benign prostate and prostate cancer

A subset of cases probed for *ASPN* expression were also dually examined by the RNAscope® Duplex Assay for *FAP* expression in stroma adjacent to benign prostate (*n* = 11) and in Gleason grade 3 (*n* = 5), Gleason grade 4 with noncribriform morphology (*n* = 8), and Gleason grade 4 with cribriform morphology (*n* = 6) prostate cancer. The percent *FAP* ^+^ cells in the stroma adjacent to Gleason grade 4 cribriform prostate cancer was highly heterogeneous among patients, with an approximate range between 5 and 50% positive (Figure 1E). Despite this interpatient heterogeneity, the collective percentage of *FAP* ^+^ cells and the expression per positive cell was significantly enriched in the tumor microenvironment adjacent to Gleason grade 4 cribriform prostate cancer compared to benign prostate (Figure 1E,F). The overall *FAP H*‐score was significantly higher in Gleason grade 4 cribriform prostate cancer compared to Gleason grade 4 prostate cancer with noncribriform morphologies, Gleason grade 3 prostate cancer, and benign prostate (Figure 1G).

Stroma was additionally analyzed for dual expression of *FAP* and *ASPN*. *ASPN* ^+^ *FAP* ^+^ cells and *ASPN* ^−^ *FAP* ^+^ cells were equally abundant at low levels in stroma adjacent to benign prostate; however, *ASPN* ^+^ *FAP* ^+^ cells were selectively enriched in the cribriform prostate tumor microenvironment compared to *ASPN* ^−^ *FAP* ^+^ cells. In contrast, *ASPN* ^*+*^ *FAP* ^−^ cells were as equally enriched as *ASPN* ^*+*^ *FAP* ^+^ cells in the microenvironment adjacent to cribriform prostate cancer (Figure 1H and supplementary material, Table S5).

### *THY1* expression in benign prostate and prostate cancer

**Figure 2:**
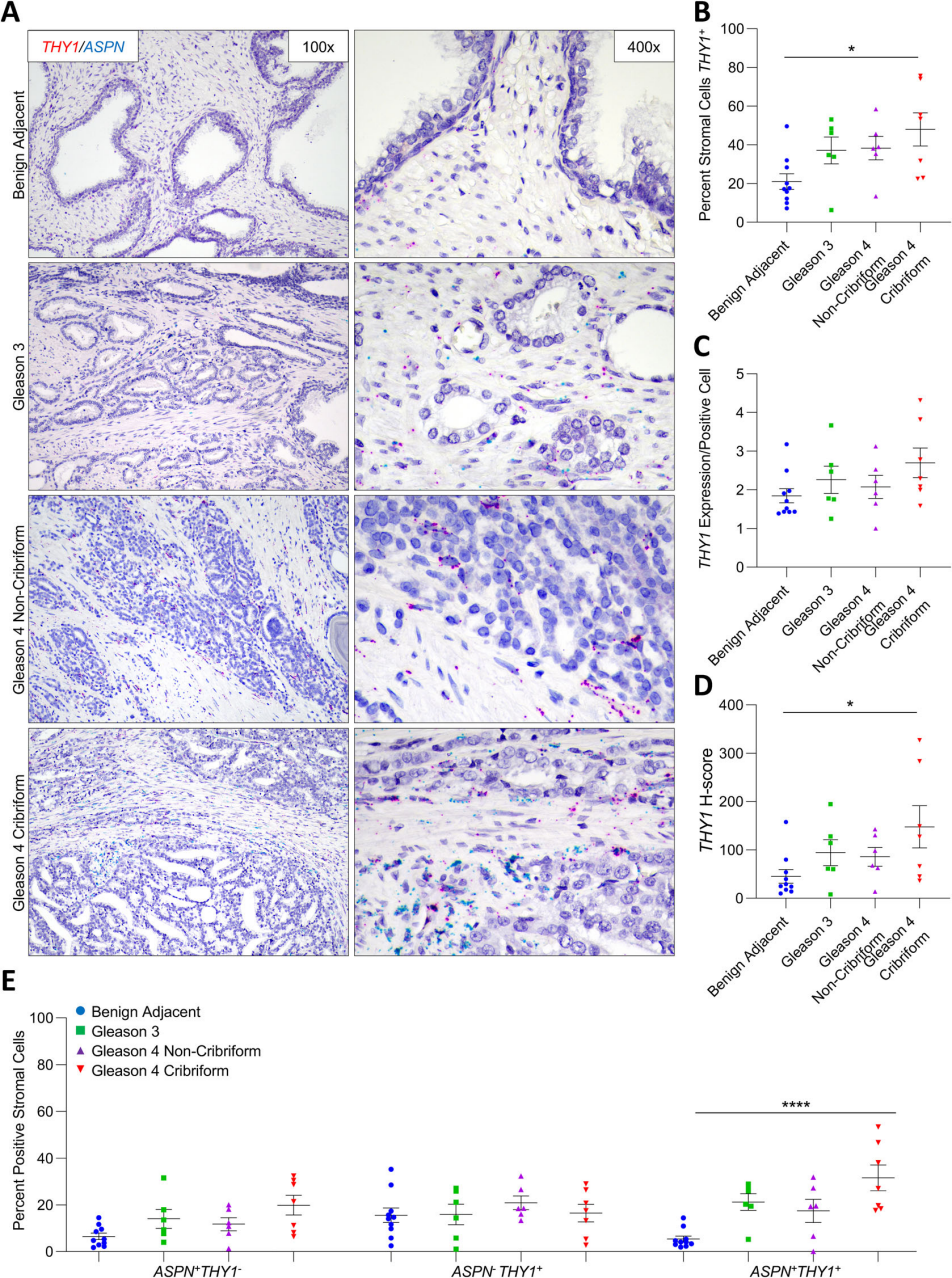
*ASPN* and *THY1* expression in cribriform prostate cancer. (A) Representative images of dual *ASPN* (blue) and *THY1* (red) mRNA expression in stroma adjacent to benign prostate (*n* = 10), Gleason grade 3 prostate cancer (*n* = 6), Gleason grade 4 prostate cancer without cribriform morphology (*n* = 6), and Gleason grade 4 prostate cancer with cribriform morphology (*n* = 7) (×400 magnification). (B) The percentage of *THY1* ^+^ stromal cells adjacent to benign prostate, Gleason grade 3 prostate cancer, Gleason grade 4 prostate cancer without cribriform morphology, and Gleason grade 4 prostate cancer with cribriform morphology. (C) The relative expression of *THY1* in *THY1* ^*+*^ stromal cells. (D) The mean *THY1 H*‐score. (E) The percent *ASPN* ^+^ *THY1* ^−^, *ASPN* ^−^ *THY1* ^+^, and *ASPN* ^+^ *THY1* ^+^ stromal cells adjacent to benign prostate, Gleason grade 3 prostate cancer, Gleason grade 4 prostate cancer without cribriform morphology, and Gleason grade 4 prostate cancer with cribriform morphology. Gene expression detected by RNAScope® and quantified with Halo® Software. Statistical analyses performed using one‐way analysis of variance with Tukey multiple comparisons. Graphs shown as mean ± SEM, *p ≤ 0.05, ****p < 0.0001.

A subset of cases probed for *ASPN* expression were also dually examined for *THY1* expression in stroma adjacent to benign prostate (*n* = 10) and in Gleason grade 3 (*n* = 6), Gleason grade 4 with noncribriform morphology (*n* = 6), and Gleason grade 4 with cribriform morphology (*n* = 7) prostate cancer. The percentage of *THY1*+ cells was significantly increased within the stroma adjacent to Gleason 4 prostate cancer with cribriform morphology compared to stroma adjacent to benign prostate glands (Figure 2A,B). Similar to *FAP*, the percentage of *THY1* ^+^ cells was heterogeneous in stroma adjacent to cribriform prostate cancer. *THY1* expression per positive cell in the cribriform prostate tumor microenvironment trended higher than in benign stroma but did not achieve the threshold for significance (Figure 2C). The overall *THY1 H*‐score remained significantly higher in stromal cells adjacent to Gleason grade 4 prostate cancer with cribriform morphology compared to stromal cells adjacent to benign glands (Figure 2D). Although the *THY1 H*‐score in Gleason grade 4 prostate cancer with cribriform architecture was elevated compared to Gleason grade 4 prostate cancer with noncribriform morphologies and Gleason grade 3 prostate cancer, this trend did not pass the threshold for significance.

Samples were additionally analyzed for dual expression of *THY1* and *ASPN*. The percentage of *ASPN* ^+^ *THY1* ^*+*^ cells was lower than *ASPN* ^*−*^ *THY1* ^*+*^ cells in the stroma adjacent to benign prostate (*p* = 0.04); however, it was the lower‐abundance *ASPN* ^+^ *THY1* ^*+*^ cells that were enriched in the cribriform prostate tumor microenvironment. *ASPN* ^*+*^ *THY1* ^*−*^ cells were not significantly enriched in the stroma adjacent to cancer (Figure 2E and supplementary material, Table S5).

### *ENG* expression in benign prostate and prostate cancer

**Figure 3:**
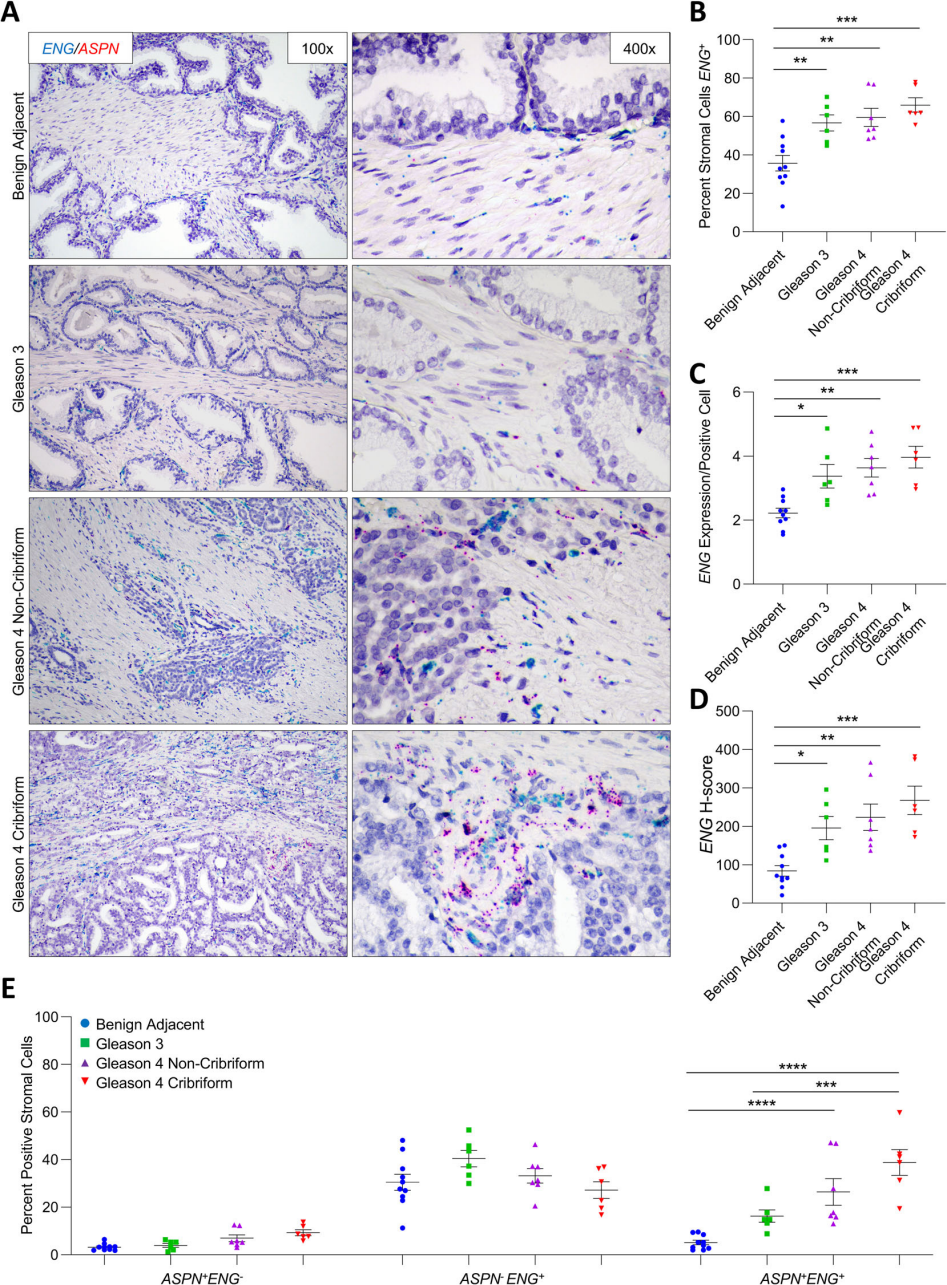
*ASPN* and *ENG* expression in cribriform prostate cancer. (A) Representative images of dual *ASPN* (red) and *ENG* (blue) mRNA expression in stroma adjacent to benign prostate (*n* = 10), Gleason grade 3 prostate cancer (*n* = 6), Gleason grade 4 prostate cancer without cribriform morphology (*n* = 7), and Gleason grade 4 prostate cancer with cribriform morphology (*n* = 6) (×400 magnification). (B) The percentage of *ENG* ^+^ stromal cells adjacent to benign prostate, Gleason grade 3 prostate cancer, Gleason grade 4 prostate cancer without cribriform morphology, and Gleason grade 4 prostate cancer with cribriform morphology. (C) The relative expression of *ENG* in *ENG* ^*+*^ stromal cells. (D) The mean *ENG H*‐score. (E) The percent *ASPN* ^+^ *ENG* ^−^, *ASPN* ^−^ *ENG* ^+^, and *ASPN* ^+^ *ENG* ^+^ stromal cells adjacent to benign prostate, Gleason grade 3 prostate cancer, Gleason grade 4 prostate cancer without cribriform morphology, and Gleason grade 4 prostate cancer with cribriform morphology. Gene expression detected by RNAScope® and quantified with Halo® Software. Statistical analyses performed using one‐way analysis of variance with Tukey multiple comparisons. Graphs shown as mean ± SEM, *p ≤ 0.05, **p ≤ 0.01, ***p ≤ 0.001, ****p < 0.0001.

*ENG* expression was also dually examined in a subset of cases probed for *ASPN* expression in stroma adjacent to benign prostate (*n* = 10) and in Gleason grade 3 (*n* = 6), Gleason grade 4 with noncribriform morphology (*n* = 7), and Gleason grade 4 with cribriform morphology (*n* = 6) prostate cancer. Both the percentage of *ENG* ^+^ stromal cells and the expression of *ENG* per positive cell were significantly increased in the stroma adjacent to prostate cancer compared to stroma adjacent to benign prostate glands, with the most marked increase in Gleason grade 4 prostate cancer with cribriform morphology (Figure 3A–C). Compared to benign adjacent stroma, the overall *ENG* ^+^ *H*‐score was significantly higher in the stroma associated with prostate cancer, independent of Gleason grade and cribriform morphology (Figure 3D).

Samples were additionally quantified for dual expression of *ASPN* and *ENG*. The percentages of *ASPN* ^+^ *ENG* ^+^ cells and *ASPN* ^*+*^ *ENG* ^−^ cells were significantly lower than *ASPN* ^*−*^ *ENG* ^*+*^ cells in the stroma adjacent to benign prostate (*p* < 0.0001); however, it was only the *ASPN* ^*+*^ *ENG* ^+^ cells that were enriched in the tumor microenvironment with the most pronounced increase in Gleason grade 4 prostate cancers containing either cribriform and noncribriform morphologies (Figure 3E and supplementary material, Table S5).

### *NT5E*, *TNC*, and *PDGFRβ* expression in benign prostate and prostate cancer

**Figure 4:**
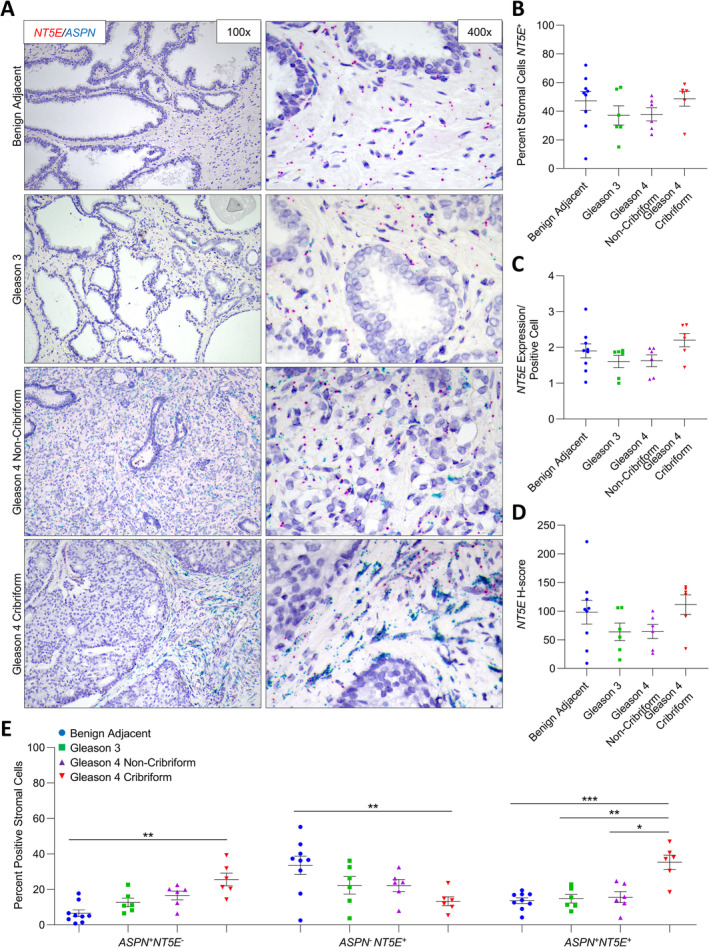
*ASPN* and *NT5E* expression in cribriform prostate cancer. (A) Representative images of dual *ASPN* (blue) and *NT5E* (red) mRNA expression in stroma adjacent to benign prostate (*n* = 9), Gleason grade 3 prostate cancer (*n* = 6), Gleason grade 4 prostate cancer without cribriform morphology (*n* = 6), and Gleason grade 4 prostate cancer with cribriform morphology (*n* = 6) (×400 magnification). (B) The percentage of *NT5E* ^+^ stromal cells adjacent to benign prostate, Gleason grade 3 prostate cancer, Gleason grade 4 prostate cancer without cribriform morphology, and Gleason grade 4 prostate cancer with cribriform morphology. (C) The relative expression of *NT5E* in *NT5E* ^*+*^ stromal cells. (D) The mean *NT5E H*‐score. (E) The percent *ASPN* ^+^ *NT5E* ^−^, *ASPN* ^−^ *NT5E* ^+^, and *ASPN* ^+^ *NT5E* ^+^ stromal cells adjacent to benign prostate, Gleason grade 3 prostate cancer, Gleason grade 4 prostate cancer without cribriform morphology, and Gleason grade 4 prostate cancer with cribriform morphology. Gene expression detected by RNAScope® and quantified with Halo® Software. Statistical analyses performed using one‐way analysis of variance with Tukey multiple comparisons. Graphs shown as mean ± SEM, *p ≤ 0.05, **p ≤ 0.01, ***p ≤ 0.001.

**Figure 5:**
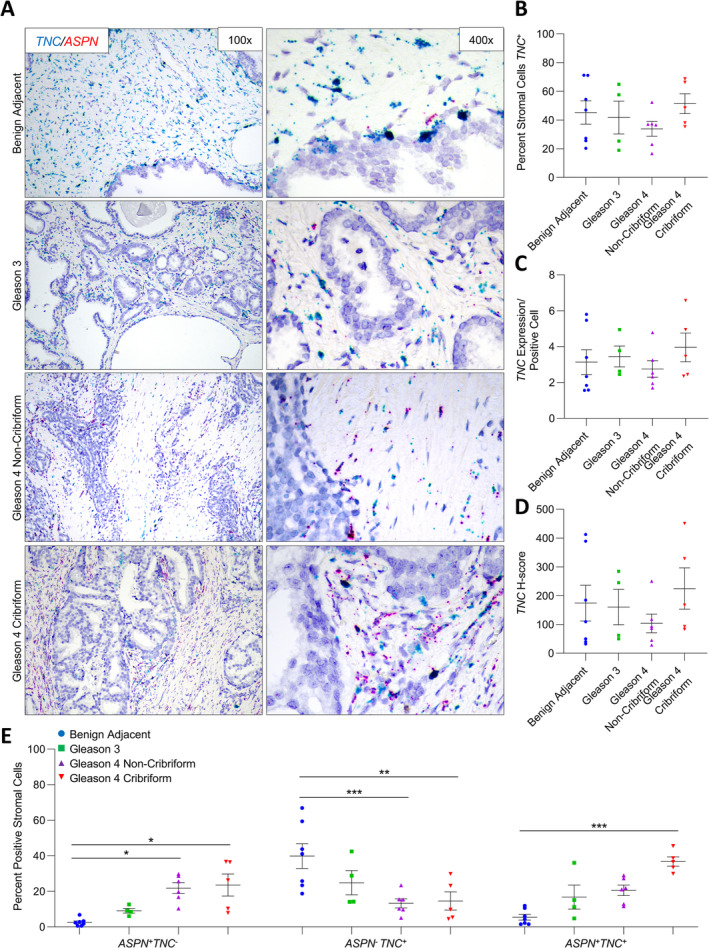
*ASPN* and *TNC* expression in cribriform prostate cancer. (A) Representative images of dual *ASPN* (red) and *TNC* (blue) mRNA expression in stroma adjacent to benign prostate (*n* = 7), Gleason grade 3 prostate cancer (*n* = 4), Gleason grade 4 prostate cancer without cribriform morphology (*n* = 6), and Gleason grade 4 prostate cancer with cribriform morphology (*n* = 5) (×400 magnification). (B) The percentage of *TNC* ^+^ stromal cells adjacent to benign prostate, Gleason grade 3 prostate cancer, Gleason grade 4 prostate cancer without cribriform morphology, and Gleason grade 4 prostate cancer with cribriform morphology. (C) The relative expression of *TNC* in *TNC* ^*+*^ stromal cells. (D) The mean *TNC H*‐score. (E) The percent *ASPN* ^+^ *TNC* ^−^, *ASPN* ^−^ *TNC* ^+^, and *ASPN* ^+^ *TNC* ^+^ stromal cells adjacent to benign prostate, Gleason 3 prostate cancer, Gleason 4 without cribriform morphology prostate cancer, and Gleason 4 with cribriform morphology prostate cancer. Gene expression detected by RNAScope® and quantified with Halo® Software. Statistical analyses performed using one‐way analysis of variance with Tukey multiple comparisons. Graphs shown as mean ± SEM, *p ≤ 0.05, **p ≤ 0.01, ***p ≤ 0.001.

**Figure 6:**
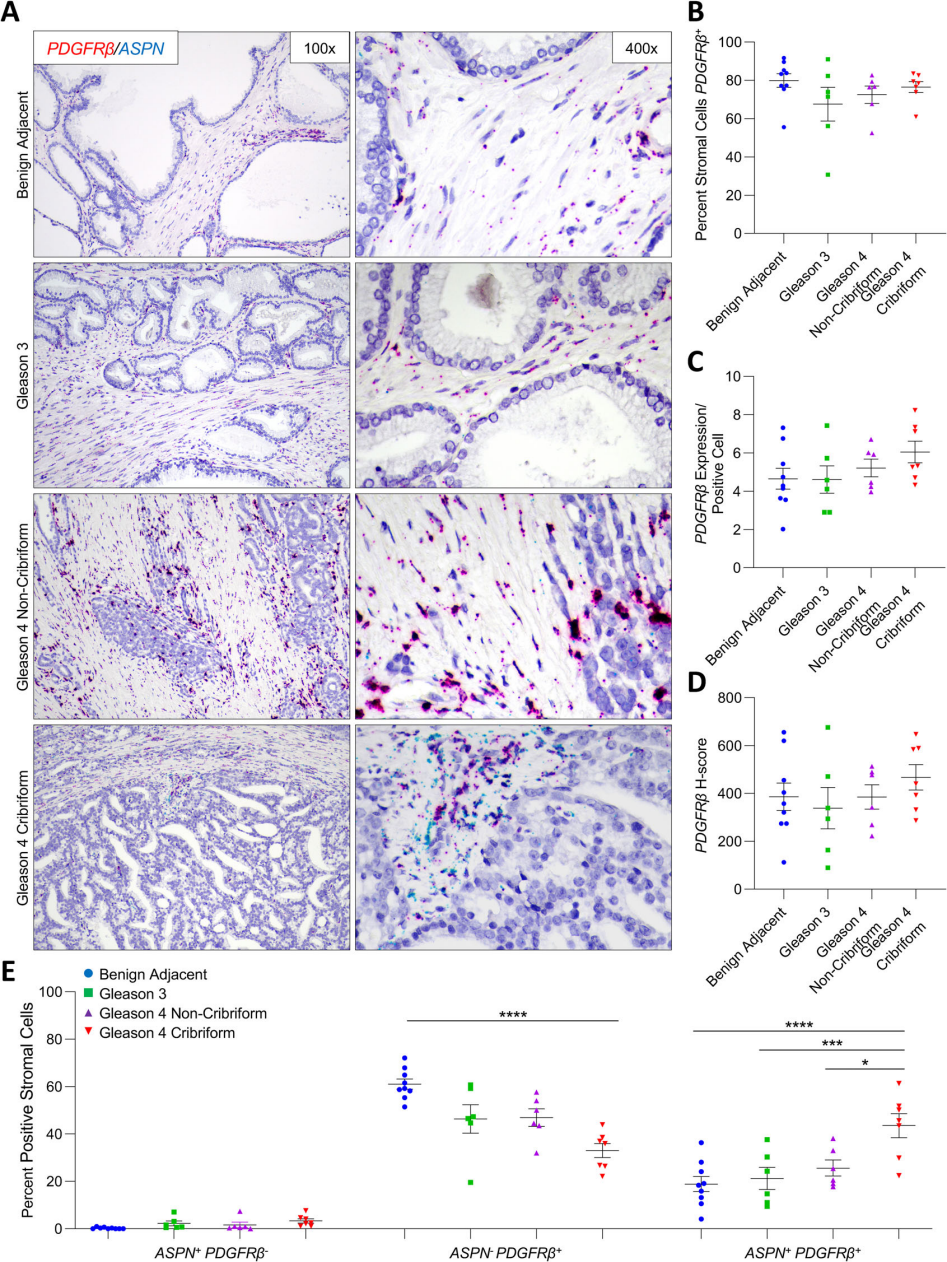
*ASPN* and *PDGFRβ* expression in cribriform prostate cancer. (A) Representative images of dual *ASPN* (blue) and *PDGFRβ* (red) mRNA expression in stroma adjacent to benign prostate (*n* = 9), Gleason grade 3 prostate cancer (*n* = 6), Gleason grade 4 prostate cancer without cribriform morphology (*n* = 6), and Gleason grade 4 prostate cancer with cribriform morphology (*n* = 7) (×400 magnification). (B) The percentage of *PDGFRβ* ^+^ stromal cells adjacent to benign prostate, Gleason grade 3 prostate cancer, Gleason grade 4 prostate cancer without cribriform morphology, and Gleason grade 4 prostate cancer with cribriform morphology. (C) The relative expression of *PDGFRβ* in *PDGFRβ* ^*+*^ stromal cells. (D) The mean *PDGFRβ H*‐score. (E) The percent *ASPN* ^+^ *PDGFRβ* ^‐^, *ASPN* ^−^ *PDGFRβ* ^*+*^, and *ASPN* ^+^ *PDGFRβ* ^+^ stromal cells adjacent to benign prostate, Gleason grade 3 prostate cancer, Gleason grade 4 prostate cancer without cribriform morphology, and Gleason grade 4 prostate cancer with cribriform morphology. Gene expression detected by RNAScope® and quantified with Halo® Software. Statistical analyses performed using one‐way analysis of variance with Tukey multiple comparisons. Graphs shown as mean ± SEM, *p ≤ 0.05, ***p ≤ 0.001, ****p < 0.0001.

*NT5E*, *TNC*, and *PDGFRβ* expression were also dually examined in a subset of cases probed for *ASPN* expression in stroma adjacent to benign prostate (*n* ≥ 7) and in Gleason grade 3 (*n* ≥ 4), Gleason grade 4 with noncribriform morphology (*n* ≥ 6), and Gleason grade 4 with cribriform morphology (*n* ≥ 5) prostate cancer. The percentages of *NT5E* ^+^, *TNC* ^+^, and PDGFR*β* ^*+*^ cells did not significantly vary between stroma adjacent to benign prostate glands and prostate cancer (Figures 4A,B, 5A,B, and 6A,B, and supplementary material, Table S5). Furthermore, *NT5E*, *TNC*, and PDGFR*β* expression per positive cell was not significantly different between benign adjacent stromal cells and prostate cancer‐adjacent stromal cells (Figure 4C, 5C, and 6C).

While the overall percentage of *NT5E* ^*+*^, *TNC* ^+^, and *PDGFRβ* ^*+*^ cells did not vary in the prostate tumor microenvironment compared to benign prostate, subsets of *NT5E* ^*+*^, *TNC* ^*+*^, and *PDGFRβ* ^*+*^ cells detected in benign prostate stroma were differentially altered in the stroma adjacent to Gleason grade 4 cribriform prostate cancer. The percentages of *ASPN* ^+^ *NT5E* ^+^ cells and *ASPN* ^*+*^ *NT5E* ^−^ cells were significantly lower than the percentage of *ASPN* ^*−*^ *NT5E* ^*+*^ cells in benign prostate stroma (*p* ≤ 0.0005); however, only the *ASPN* ^+^ *NT5E* ^−^ cells and the *ASPN* ^+^ *NT5E* ^+^ cells detected in the stroma adjacent to benign prostate glands were enriched in the cribriform prostate tumor microenvironment, while the more abundant *ASPN* ^*−*^ *NT5E* ^*+*^ cells were significantly decreased (Figure 4E). This corresponding loss of *ASPN* ^*−*^ *NT5E* ^*+*^ cells and gain of *ASPN* ^+^ *NT5E* ^+^ cells without a net change in total *NT5E* ^+^ cells in the stroma adjacent to cribriform prostate cancer compared to benign adjacent stroma suggests that *ASPN* was induced in *NT5E* ^+^ cells in the cribriform prostate tumor microenvironment. Findings were similar for *TNC* with a significant increase in the cribriform prostate tumor microenvironment of the lesser abundant *ASPN* ^*+*^ *TNC* ^−^ cells and *ASPN* ^*+*^ *TNC* ^+^ cells in benign prostate stroma and a corresponding significant decrease in the more abundant *ASPN* ^*−*^ *TNC* ^+^ cells in benign prostate stroma (Figure 5E). Nearly all cells in both benign prostate stroma and prostate cancer stroma were *PDGFRβ* ^*+*^. *PDGFRβ* ^*+*^ cells shifted from being predominately *ASPN* ^−^ in benign prostate stroma to being comparably *ASPN* ^−^ and *ASPN* ^+^ in the cribriform prostate tumor microenvironment (Figure 6E and supplementary material, Table S5). This suggests that ASPN was induced in a subset of *PDGFRβ* ^*+*^ cells in the cribriform prostate microenvironment.

## Discussion

Cribriform morphology is a distinct subtype of Gleason grade 4 prostate cancer that has been shown to be associated with worse outcomes [6, 7, 13, 59]. The mechanisms underlying this aggressive phenotype are not fully understood. Multiple studies have focused on delineating the genetic and molecular pathways that differentiate prostate cancer with cribriform morphology from other prostate cancers including other morphologies of Gleason grade 4 [13, 19, 20, 21, 59, 60]. Less is understood about the features that are characteristic to the microenvironment of cribriform prostate cancer. Due to the cribriform architecture, most cribriform tumor cells do not directly contact the stroma; however, cribriform glands are often surrounded by densely packed stromal cells. How fibroblast subtypes associated with invasive cribriform prostate carcinoma compare to fibroblasts associated with other prostate cancers, including other Gleason grade 4 morphologies, is not known. Identification of specific fibroblast cell types in the cribriform tumor microenvironment will be necessary to determine their impact on cribriform cancer development and progression.

This study demonstrates the robust heterogeneity of fibroblasts in the prostate tumor microenvironment and suggests that fibroblast subtypes may vary with Gleason grade and morphology. The findings presented here support the hypothesis that a distinct repertoire of fibroblasts, present at low levels in benign prostate stroma, may be selectively enriched in Gleason grade 4 cribriform prostate cancer compared to Gleason grade 4 noncribriform prostate cancer or Gleason grade 3 prostate cancer. Overall, *FAP* ^+^ cells and *THY1* ^+^ cells were enriched in the microenvironment of Gleason grade 4 cribriform prostate cancer compared to stroma adjacent to benign prostate. However, only the subset of *FAP* ^+^ cells and *THY1* ^+^ cells that were also *ASPN* ^+^ accounted for this increase. Some cell types enriched in the cribriform tumor microenvironment were also increased in other Gleason grade 4 morphologies. The percentage of *ENG* ^+^ cells was increased in the tumor microenvironment, independent of Gleason grade or morphology. While low in benign adjacent stroma, *ENG* ^*+*^ *ASPN* ^+^ cells largely accounted for this increase in the cancer‐adjacent stroma. Thus, it is likely that low‐abundance tissue‐resident fibroblasts are expanded in multiple tumor grades and morphologies, while others are distinctly amplified in the cribriform tumor microenvironment.

This study also supports the premise that distinct gene expression programs may be induced in existing fibroblasts adjacent to Gleason grade 4 cribriform prostate cancer compared to other Gleason grade 4 morphologies. In contrast to *FAP* ^+^ cells and *THY1* ^+^ cells, the overall percentages of *NT5E* ^+^ cells, *TNC* ^+^ cells, and *PDGFRβ* ^+^ cells were not altered in the tumor microenvironment compared to benign prostate stroma. However, *NT5E* ^+^ cells, *TNC* ^+^ cells, and *PDGFRβ* ^+^ cells shifted from *ASPN* ^−^ to *ASPN* ^+^ in the cribriform tumor microenvironment. As *ASPN* expression was increased in cribriform prostate cancer, this collectively suggests that *ASPN* expression was induced in *NT5E* ^+^ *ASPN* ^−^ fibroblasts, *TNC* ^+^ *ASPN* ^−^ fibroblasts, and *PDGFRβ* ^+^ *ASPN* ^−^ fibroblasts in the cribriform tumor microenvironment. Induced gene expression in subsets of fibroblasts may also occur to a lesser extent in lower Gleason grades and in other Gleason grade 4 morphologies. *TNC* ^+^ cells trended from *ASPN* ^−^ in benign adjacent stroma to *ASPN* ^+^ in stroma adjacent to Gleason grade 3 and Gleason grade 4 with noncribriform morphology prostate cancer. These findings suggest that cribriform prostate cancer may induce both a shared as well as a distinct gene expression profile in the tumor microenvironment that differentiates cribriform from other Gleason grade 4 morphologies.

Improved identification of the fibroblast subtypes associated with cribriform prostate cancer will impact our understanding of how the tumor microenvironment regulates cribriform growth and progression. In this study, fibroblast subtypes enriched in the cribriform tumor microenvironment were *ASPN* ^+^. ASPN expression in CAFs has been shown to impact the tumor microenvironment, to enhance cellular invasion, and to promote metastatic progression [29, 32, 33, 61]. These findings show that *ASPN* ^+^ fibroblasts amplified in the cribriform microenvironment were equally *FAP* ^+^ and *FAP* ^−^. Due to its expression in CAFs, role in immune suppression [39], and membrane‐bound extracellular localization, FAP may be an ideal target for imaging and therapies [41, 42, 43, 44, 45, 46]. Targeting FAP would additionally target a subpopulation of ASPN^+^ cells. *ASPN* ^*+*^ cells enriched in cribriform prostate cancer were also *THY1* ^+^. Interestingly, ASPN and THY1 have both been shown to impact cancer stem cells [29, 48]. Similar to THY1, *ENG* ^+^ *ASPN* ^+^ fibroblasts were also elevated in cribriform prostate cancer. Both ENG and ASPN are associated with adverse outcomes and may promote the progression of aggressive prostate cancer [32, 49, 50, 51]. While the overall expression of *NT5E*, *TNC*, and *PDGFRβ* did not change in the tumor microenvironment, *ASPN* expression was altered in these cells. Studies are currently assessing *NT5E*, *TNC*, and *PDGFRβ* as candidates for therapeutic targeting [52, 53, 54, 55]. To date, strategies targeting ASPN directly have not been reported, and therefore, therapies directed to these other markers may be an ideal way to target *ASPN* ^+^ fibroblasts.

There are limitations to this study. For optimal analysis by RNAScope®, it is recommended that FFPE tissue be processed within 1 year of collection. Consequently, long‐term follow up was not available for the patients in this study. In addition, the RNAScope® assay is only optimized for two markers for chromogenic analyses, which limits multiplexing. Future studies will examine larger cohorts using fluorescent‐based assays that allow for multiplexing of more markers or unbiased methods such as single‐cell RNA sequencing to fully characterize the heterogeneity in the CAF transcriptome. Additional future studies will use patient‐derived CAFs to elucidate their functional role in cancer progression and therapy resistance.

In summary, the findings from this study highlight the substantial heterogeneity in CAF subtypes enriched in prostate cancer. This study further supports that a distinct population of rare fibroblasts in benign prostate stroma are selectively enriched in the cribriform prostate tumor microenvironment. In addition, distinct gene expression programs may be induced in existing fibroblasts adjacent to cribriform prostate cancer. It is likely that cribriform prostate cancer has a unique tumor microenvironment that distinguishes it from other Gleason pattern 4 morphologies and other Gleason grades.

## Author contributions statement

PJH, JBG, TLL, KRS and GAG conceived and designed the study. ABH, BLR, EMW, CT, HYW, JBG, GAG, TLL and PJH acquired and analyzed data. All authors interpreted data and prepared the manuscript.

## Supporting information


**Supplementary materials and methods**
**Figure S1.** Validation of controls utilized in RNAscope®
**Figure S2.** Comparison of IHC and RNAscope® in patient samples
**Table S1.** Target probe information and experimental design
**Table S2.** Incubation steps for cell pellets and tissue sections
**Table S3.** Antibody information and IHC conditions
**Table S4.** Patient characteristics
**Table S5.** Change in percent positive stromal cells adjacent to prostate cancer compared to benign prostate
