# Supplementary material for: A distinct repertoire of cancer‐associated fibroblasts is enriched in cribriform prostate cancer
Source: J Pathol Clin Res. 2021 Feb 18;7(3):271–86. doi: 10.1002/cjp2.205 (PMC8073007; doi:10.1002/cjp2.205)
Supplement: Supplementary file 1 — Supplementary materials and methods Figure S1. Validation of controls utilized in RNAscope® Figure S2. Comparison of IHC and RNAscope® in patient samples Table S1. Target probe information and experimental design Table S2. Incubation steps for cell pellets and tissue sections Table S3. Antibody information and IHC conditions Table S4. Patient characteristics Table S5. Change in percent positive stromal cells adjacent to prostate cancer compared to benign prostate [file CJP2-7-271-s001.docx]

**A distinct repertoire of cancer-associated fibroblasts is enriched in cribriform prostate cancer**

AB Hesterberg *et al*. *J Pathol Clin Res* DOI: 10.1002/cjp2.205

**Supplementary Material**

**Supplementary Materials and Methods**

**Figure S1.** Validation of controls utilized in RNAscope®

**Figure S2.** Comparison of IHC and RNAscope® in patient samples

**Table S1.** Target probe information and experimental design

**Table S2.** Incubation steps for cell pellets and tissue sections

**Table S3.** Antibody information and IHC conditions

**Table S4.** Patient characteristics

**Table S5.** Change in percent positive stromal cells adjacent to prostate cancer compared to benign prostate

**Supplementary Material and Methods**

**Dual RNA *in situ* hybridization**

Patient FFPE whole tissue sections (4µm) from radical prostatectomy were analyzed for expression of *ASPN* in combination with the following markers: *THY1*, *NT5E*, *ENG*, *PDGFRβ*, *FAP*, and *TNC* (supplementary material, Table S1) using the RNAscope® 2.5 HD Duplex Assay by Advanced Cell Diagnostics (ACD, Newark, CA, USA, 322430) according to the manufacturer’s recommendations. In brief, FFPE slides were baked and then deparaffinized in xylene followed incubation in 100% ethanol. Slides were removed, air dried, and then covered with H_2_O_2_. Using a preheated steamer (Oster, Model No. 5712), slides were submerged into boiling distilled water and then boiled in Target Retrieval Reagent. Following incubation, slides were rinsed with distilled water, transferred to 100% ethanol, and then air dried. Slides were treated with Protease Plus and incubated at 40ºC in a prewarmed HybEZ II oven (ACD 321710). The C1 and C2 target probes were warmed and then combined in a 50:1 ratio of C1 to C2. Slides were removed from oven, decanted, treated with probe solution and baked at 40ºC. Slides were then washed in Wash Buffer. Slides were stored in 5X SSC solution overnight at room temperature. The next day, slides were washed in Wash Buffer. Red signal was developed by incubating the tissue sections in Amp 1, Amp 2, Amp 3, and then Amp 4 at 40ºC. Slides were washed with Wash Buffer between each amplification step. Tissue sections were then incubated with Amp 5 and Amp 6 in a humidifying chamber at room temperature. Slides were washed with 1X Wash Buffer between each step. Red signal was detected by mixing a 1:60 ratio of Fast Red-B to Fast Red-A and incubating each section with red solution at room temperature in a humidifying chamber. Slides were decanted and washed in Wash Buffer with gentle agitation. Green signal was developed by incubating the tissue sections in Amp 7 and Amp 8 at 40ºC. Slides were washed with 1X Wash Buffer between each step. Tissue sections were then incubated with Amp 9 and Amp 10 in a humidifying chamber at room temperature. Slides were washed with Wash Buffer between amplification steps. Green signal was detected by mixing a 1:50 ratio of Fast Green-B to Fast Green-A. Slides were treated with green solution and incubated at room temperature in a humidifying chamber. Slides were removed, decanted and briefly submerged in 1X Wash Buffer. Slides were then rinsed in distilled water before staining with Hematoxylin. Slides were mounted with VectaMount (Vector, 64742-48-9) and cover slipped (supplementary material, Table S2).


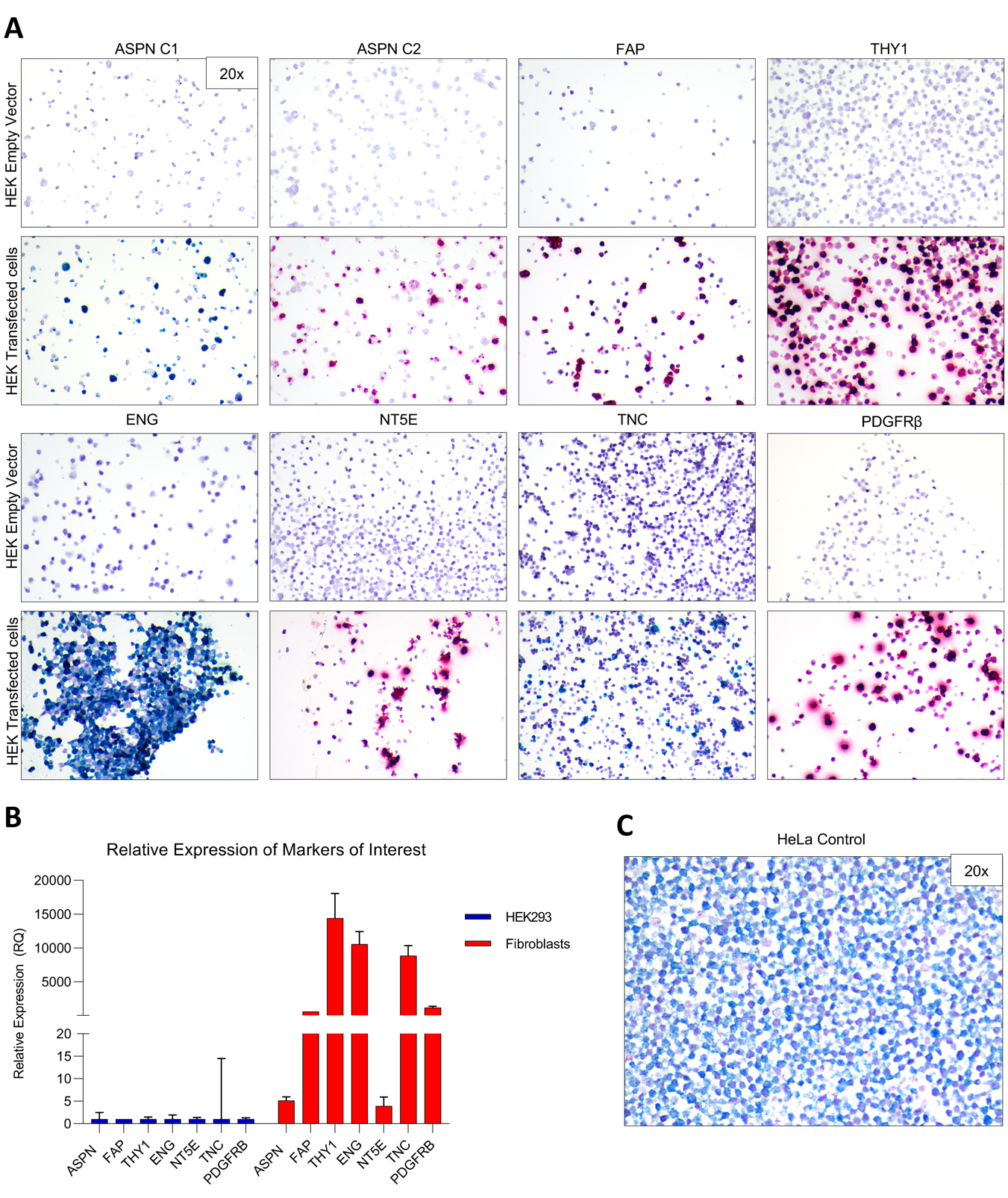


**Figure S1. Validation of controls utilized in RNAscope®.** (A) Representative images of dual RNAscope® control slides to validate probe specificity (20x magnification). HEK-293 cells were transfected with plasmids of interest and empty vectors to generate positive and negative cell pellets. Control slides were run in tandem with patient samples. (B) Relative gene expression of fibroblast markers in HEK-293 cells compared to cancer-associated fibroblasts as measured by RT-qPCR. (C) Representative image of dual RNAscope® HeLa control slide to validate the RNAscope® technique (20x magnification).


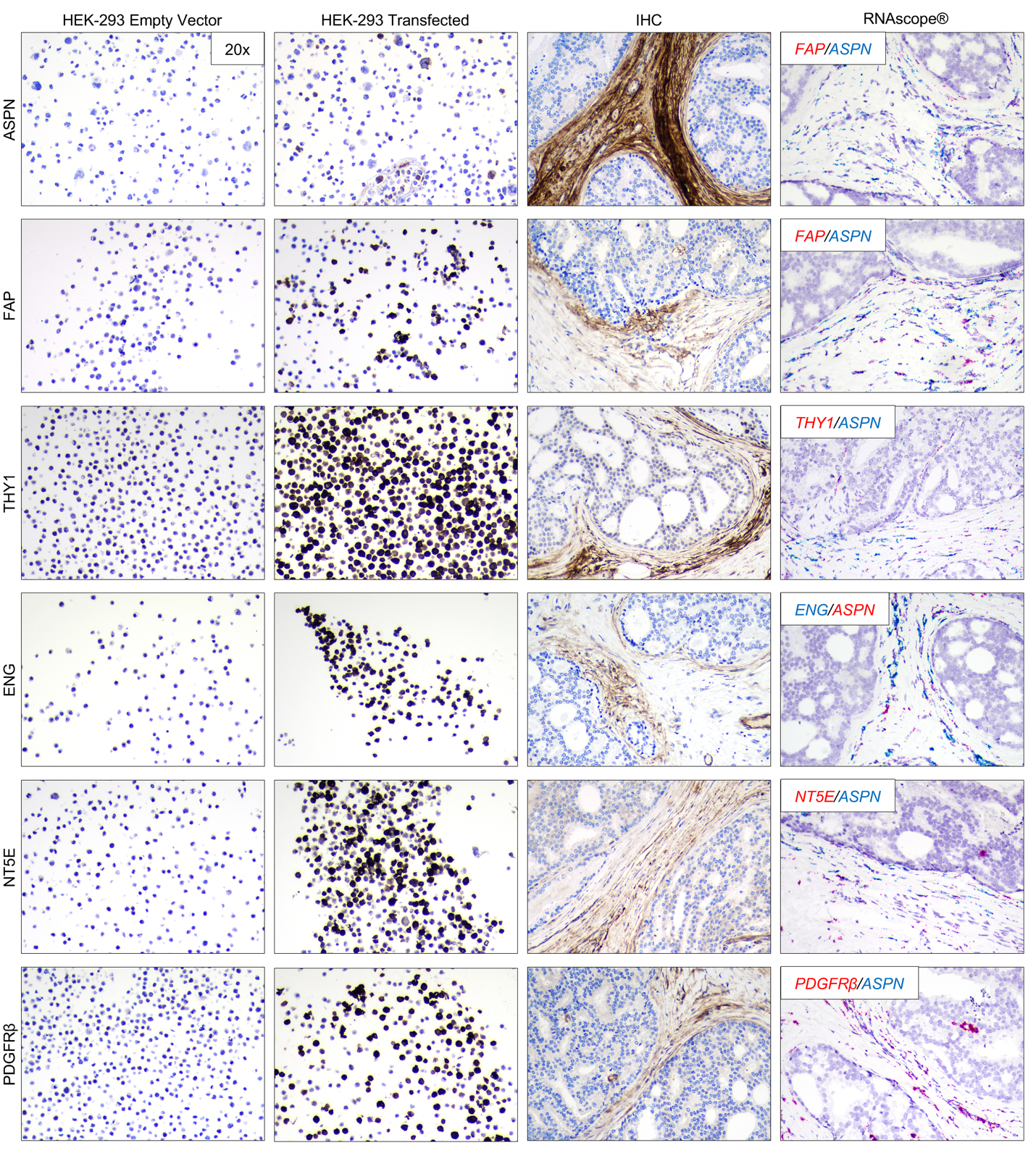


**Figure S2. Comparison of IHC and RNAscope® in patient samples.** Representative images of IHC control slides to validate antibody specificity (20x magnification). HEK-293 cells were transfected with plasmids of interest and empty vectors to generate positive and negative cell pellets. Representative images of IHC and RNAscope® in matched patient samples (20x magnification). Limited ASPN staining in the cell pellet control may be due to its secretion and loss during cell processing. Control slides were run in tandem with patient samples.

| **Table S1. Target probe information and experimental design** | | | | |
| --- | --- | --- | --- | --- |
| **Markers Analyzed** | **Probe C1** | **Probe C2** | **C2:C1 Ratio** | **OriGene cDNA used for HEK293 transfection** |
| ***FAP/ASPN*** | Hs-ASPN (ACD 404481) | Hs-FAP (ACD 411971-C2) | 50:1 | *FAP* (RG204692) |
| ***THY1/ASPN*** | Hs-ASPN (ACD 404481) | Hs-THY1 (ACD 430611-C2) | 50:1 | *THY1* (RG209458)  *ASPN* (RC209353) |
| ***ENG/ASPN*** | Hs-ENG (ACD 484111) | Hs-ASPN (404481-C2) | 50:1 | *ENG* (RG226069) |
| ***NT5E/ASPN*** | Hs-ASPN (ACD 404481) | Hs-NT5E (ACD 437931-C2) | 50:1 | *NT5E* (RG209568) |
| ***TNC/ASPN*** | Hs-TNC (ACD 420771) | Hs-ASPN (ACD 404481-C2) | 50:1 | *TNC* (RG215251) |
| ***PDGFRβ/ASPN*** | Hs-ASPN (ACD 404481) | Hs-PDGFRβ (ACD 548991-C2) | 50:1 | *PDGFRβ* (RG206377) |
| **HeLa Control (ACD 310045)** | Positive Probe (ACD 321641) | Negative Probe (ACD 320751) | 1:1 | N/A |

| **Table S2. Incubation steps for cell pellets and tissue sections** | | | |
| --- | --- | --- | --- |
| **Reagent** | **Incubation Temperature** | **Cell Pellet Incubation Time (min)** | **Tissue Section Incubation time (min)** |
| **H_2_O_2_** | RT | 10 | 10 |
| **1X Target Retrieval** | 99°C | 8 | 15 |
| **Protease Plus** | 40°C | 15 | 30 |
| **Probe mixture** | 40°C | 120 | 120 |
| **Amp 1** | 40°C | 30 | 30 |
| **Amp 2** | 40°C | 15 | 15 |
| **Amp 3** | 40°C | 30 | 30 |
| **Amp 4** | 40°C | 15 | 15 |
| **Amp 5** | RT | 30 | 30 |
| **Amp 6** | RT | 15 | 15 |
| **Red Signal** | RT | 10 | 10 |
| **Amp 7** | 40°C | 15 | 15 |
| **Amp 8** | 40°C | 30 | 30 |
| **Amp 9** | RT | 30 | 30 |
| **Amp 10** | RT | 15 | 15 |
| **Green signal** | RT | 10 | 10 |

| **Table S3. Antibody information and IHC conditions** | | | | | | | |
| --- | --- | --- | --- | --- | --- | --- | --- |
| **Antibody**  **Target** | **Antibody**  **Company** | **Antibody**  **Reference Number** | **Antigen Retrieval** | **Antibody**  **Dilution** | **Secondary**  **Antibody** | **OriGene cDNA used for HEK-293 transfection** |  |
| **ASPN** | Sigma | HPA008435 | Dako Target Retrieval Solution EDTA pH9  (S236884-2) | 1:400 | Rabbit | ASPN (RC209353) |  |
| **FAP** | Abcam | ab227703 | Dako Target Retrieval Solution EDTA pH9  (S236884-2) | 1:100 | Rabbit | FAP (RG204692) |  |
| **THY1** | Abcam | ab92574 | Dako Target Retrieval Solution EDTA pH9  (S236884-2) | 1:100 | Rabbit | THY1 (RG209458) |  |
| **ENG** | Sigma | HPA067440 | Dako Target Retrieval Solution, Citrate pH6.1 (S16999) | 1:500 | Rabbit | ENG (RG226069) |  |
| **NT5E** | Cell Signaling Technology | 13160 | Invitrogen  Antigen Retrieval Solution high pH  (00-4956-58) | 1:200 | Rabbit | NT5E (RG209568) |  |
| **PDGFRβ** | Cell Signaling Technology | 3169 | Dako Target Retrieval Solution EDTA pH9  (S236884-2) | 1:100 | Rabbit | PDGFRβ (RG206377) |  |

| **Table S4. Patient characteristics** | |
| --- | --- |
| **Median age (years; range) (n=21)** | 61 (48-73) |
| **Race (n=21)** |  |
| Caucasian | 17 (81%) |
| African American | 2 (9.5%) |
| Other | 2 (9.5%) |
| **Pathologic stage (AJCC TNM 8^th^ Edition; n=19)** |  |
| pT2N0 | 5 (26%) |
| pT3aN0 | 6 (32%) |
| pT3bN0 | 7 (37%) |
| pN1 | 1 (5%) |
| **Pathologic Grade (n=21)** |  |
| Grade Group 1 | 0 (0%) |
| Grade Group 2 | 5 (24%) |
| Grade Group 3 | 13 (62%) |
| Grade Group 4 (4+4=8) | 0 (0%) |
| Grade Group 5 (4+5=9) | 3 (14%) |
| **Positive surgical margins (n=21)** | 8 (38%) |

| **Table S5. Change in percent positive stromal cells adjacent to prostate cancer compared to benign prostate** | | | |
| --- | --- | --- | --- |
| **Marker** | **Gleason 3** | **Gleason 4**  **Non-cribriform** | **Gleason 4**  **Cribriform** |
| *ASPN^+^* | + | ++ | +++ |
| *FAP^+^* | ns | ns | ++ |
| *THY1^+^* | ns | ns | ++ |
| *ENG^+^* | ++ | ++ | ++ |
| *NT5E^+^* | ns | ns | ns |
| *TNC^+^* | ns | ns | ns |
| *PDGFRβ^+^* | ns | ns | ns |
|  |  |  |  |
| *ASPN^+^FAP^-^* | ns | ns | ++ |
| *FAP^+^ASPN^-^* | ns | ns | ns |
| *ASPN^+^FAP^+^* | ns | ns | ++ |
|  |  |  |  |
| *ASPN^+^THY1^-^* | ns | ns | ns |
| *THY1^+^ASPN^-^* | ns | ns | ns |
| *ASPN^+^THY1^+^* | ns | ns | ++ |
|  |  |  |  |
| *ASPN^+^ENG^-^* | ns | ns | ns |
| *ENG^+^ASPN^-^* | ns | ns | ns |
| *ASPN^+^ENG^+^* | ns | ++ | +++ |
|  |  |  |  |
| *ASPN^+^NT5E^-^* | ns | ns | ++ |
| *NT5E^+^ASPN^-^* | ns | ns | -- |
| *ASPN^+^NT5E^+^* | ns | ns | ++ |
|  |  |  |  |
| *ASPN^+^TNC^-^* | ns | ++ | ++ |
| *TNC^+^ASPN^-^* | ns | -- | -- |
| *ASPN^+^TNC^+^* | ns | ns | ++ |
|  |  |  |  |
| *ASPN^+^PDGFRβ^-^* | ns | ns | ns |
| *PDGFRβ^+^ASPN^-^* | ns | ns | -- |
| *ASPN^+^PDGFRβ^+^* | ns | ns | ++ |
|  |  |  |  |
| Abbreviation: ns, not significant | | | |
